# Restored and remnant Banksia woodlands elicit different foraging behavior in avian pollinators

**DOI:** 10.1002/ece3.7946

**Published:** 2021-07-27

**Authors:** Alison L. Ritchie, Carole P. Elliott, Elizabeth A. Sinclair, Siegfried L. Krauss

**Affiliations:** ^1^ School of Biological Science The University of Western Australia Crawley WA Australia; ^2^ Kings Park Science Department of Biodiversity, Conservation and Attractions Kings Park and Botanic Garden Kings Park WA Australia

**Keywords:** aggression, network analysis, plant–pollinator, pollination services, Pollinator Movement Index, restoration

## Abstract

Pollinators and the pollination services they provide are critical for seed set and self‐sustainability of most flowering plants. Despite this, pollinators are rarely assessed in restored plant communities, where their services are largely assumed to re‐establish. Bird–pollinator richness, foraging, and interaction behavior were compared between natural and restored Banksia woodland sites in Western Australia to assess their re‐establishment in restored sites. These parameters were measured for natural communities of varying size and degree of fragmentation, and restored plant communities of high and low complexity for three years, in the summer and winter flowering of *Banksia attenuata* and *B. menziesii*, respectively. Bird visitor communities varied in composition, richness, foraging movement distances, and aggression among sites. Bird richness and abundance were lowest in fragmented remnants. Differences in the composition were associated with the size and degree of fragmentation in natural sites, but this did not differ between seasons. Restored sites and their adjacent natural sites had similar species composition, suggesting proximity supports pollinator re‐establishment. Pollinator foraging movements were influenced by the territorial behavior of different species. Using a network analysis approach, we found foraging behavior varied, with more frequent aggressive chases observed in restored sites, resulting in more movements out of the survey areas, than observed in natural sites. Aggressors were larger‐bodied Western Wattlebirds (*Anthochaera chrysoptera*) and New Holland Honeyeaters (*Phylidonyris novaehollandiae*) that dominated nectar resources, particularly in winter. Restored sites had re‐established pollination services, albeit with clear differences, as the degree of variability in the composition and behavior of bird pollinators for Banksias in the natural sites created a broad completion target against which restored sites were assessed. The abundance, diversity, and behavior of pollinator services to remnant and restored Banksia woodland sites were impacted by the size and degree of fragmentation, which in turn influenced bird–pollinator composition, and were further influenced by seasonal changes between summer and winter. Consideration of the spatial and temporal landscape context of restored sites, along with plant community diversity, is needed to ensure the maintenance of the effective movement of pollinators between natural remnant woodlands and restored sites.

## Graphical abstract

Restored sites had re‐established pollinator services, albeit with clear differences. The abundance, diversity, and behavior of bird‐pollinator services to remnant and restored Banksia woodland sites were impacted by the size and degree of fragmentation, which in turn influenced bird composition, and were further influenced by seasonal changes between summer and winter.

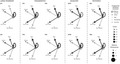

## 1 INTRODUCTION

The pollination of flowering plants by animals is a fundamental ecosystem process in terrestrial ecosystems. More than 349,000 animal species forage on flowers worldwide, with over 87% of angiosperms relying on animal‐mediated pollination for sexual reproduction and genetic recombination (Kearns & Inouye, 1997; Ollerton, 2017; Ollerton et al., 2011). The behavior of pollinators during pollination has fundamental consequences for plant mating and determines the maximum frequency and diversity of mating opportunities (Harder & Barrett, 1996; Minnaar et al., 2019; Wessinger, 2020). There is ample evidence that habitat loss, alteration, and disturbance can negatively impact pollinators and plant–pollinator interactions (Bennett et al., 2020). Impacts on pollinators and their foraging behavior can result in pollen limitation and reduced seed set for the plants dependent on their pollination services (Eckert et al., 2010; Ratto et al., 2018).

Despite this critical role, plant–pollinator relationships are rarely considered in ecosystem restoration (Cariveau et al., 2020; Forup et al., 2008; Frick et al., 2014; Menz et al., 2011; Munro et al., 2011; Williams, 2011). Instead, ecological restoration has largely focused on plant species richness and habitat structure and, consequently, restoration success has been typically measured against the achievements of these structural properties (Ruiz‐Jaén et al., 2005). Many “non‐target” animal species, such as pollinators, are assumed to passively colonize restored areas (Catterall, 2018; Williams, 2011). However, attributes of restored ecosystems, such as vegetation structure, plant species composition, density of reproductive plants, and degree of geographic isolation, can influence pollinator abundance, diversity, and behavior, ultimately affecting plant fitness (Cariveau et al., 2020). Therefore, there is a need to measure, manage, and promote ecosystem functionality in restored sites, by extending the emphasis from plant establishment to self‐sustaining populations, where pollinator community interactions are also assessed (Cariveau et al., 2020). However, a major challenge in determining the restoration of plant–pollinator interactions is the requirement to benchmark measures against natural systems, which are inherently complex and variable (Moreno‐Mateos et al., 2020).

The Southwest Australian Floristic Region (SWAFR; Hopper & Gioia, 2004) has the highest recorded frequency of vertebrate‐pollinated species in the world (Krauss et al., 2017; Phillips et al., 2010). Generalist nectivorous birds (honeyeaters) of the Meliphagidae family are the most numerous and species‐rich group of avian pollinators in Australia with ca 180 spp., of which half are native (Krauss et al., 2017), and 17 species recorded in the SWAFR (Higgins, 2006). Experimental exclusion of them from the flowers they visit often results in substantially lower fruit set (Ayre et al., 2020; Ramsey & Vaughton, 1991; Wooller & Wooller, 2001).

Pollinators typically forage optimally, whereby energetic costs during foraging are minimized by moving short distances between flowers, often between near‐neighboring plants and probing several flowers sequentially (Pyke, 1984). These foraging behaviors have important implications for plant mating (Krauss et al., 2017). Short pollinator flights may limit the extent of pollen dispersal, resulting in geitonogamy, leptokurtic pollen dispersal, bi‐parental inbreeding, low paternal diversity, and local genetic neighborhoods (Harder & Barrett, 1996; Krauss et al., 2017). However, behavioral differences in species dominance relations can result in aggressive chases between honeyeaters (Armstrong, 1991; Ford, 1979; McFarland, 1986; Ramsay, 1989) and increase pollinator flights. This increases pollen carryover and the frequency of long‐distance pollen dispersal events within and among plant populations (Phillips et al., 2014; Wessinger, 2020).

This study assessed bird–pollinator behavior from the standpoint of delivering pollinator services to two numerically dominant species in Banksia woodlands of SWAFR, *Banksia attenuata* R.Br. and *Banksia menziesii* R.Br. (Proteaceae), among multiple natural and restored sites. We conducted a novel assessment of the establishment of bird–pollinator services in restored sites, whilst addressing the inherent variability of these relationships among natural sites. Specifically, the following questions were addressed: (a) Does bird community composition and diversity differ among remnant, fragmented, and restored sites, and between *Banksia* species? (b) Do the foraging movements of birds on plants differ among remnant, fragmented, and restored sites? (c) Do aggressive bird interactions differ among remnant, fragmented, and restored sites? and (d) Are there seasonal differences in the provision of pollination services by bird‐pollinator visitation? The implications for pollinator services in these altered landscapes are discussed.

## 2 MATERIALS AND METHODS

### 2.1 Study sites

**FIGURE 1:**
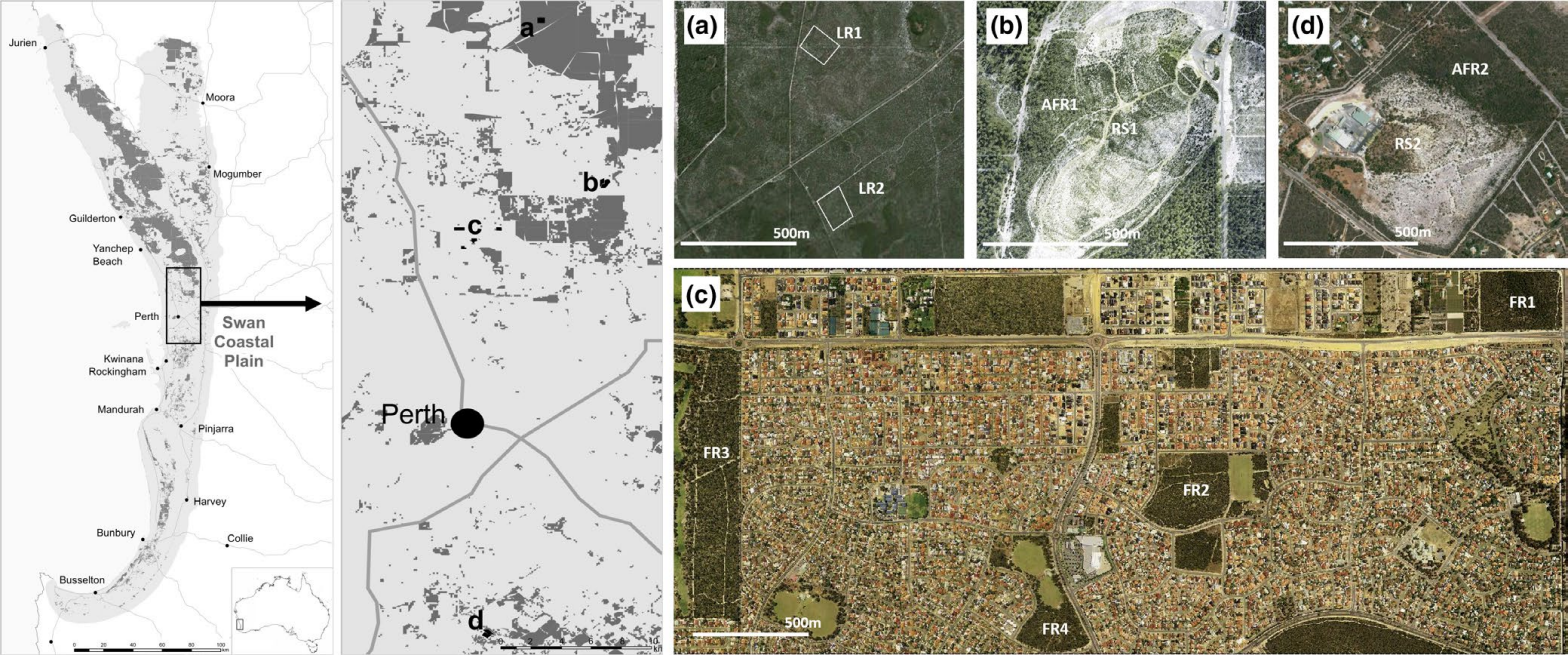
Study site locations along the Swan Coastal Plain, Western Australia. First two panels indicate pre‐European distribution of Banksia woodlands (light gray) and distribution as of 2015 (dark gray) (Environmental Resources Information Network, 2016) with site localities; (a) large connected remnant sites LR1 (Neaves North) and LR2 (Neaves South); (b) adjacent fragmented woodland site AFR1 (Northern Adjacent) and restored site RS1 (Northern Restored); (c) fragmented remnant woodland sites; FR1 (Hepburn Park), FR2 (Highview Park), FR3 (Marangaroo Conservation Reserve), and FR4 (Paloma Park); and (d) adjacent fragmented woodland site AFR2 (Southern Adjacent) and restored site RS2 (Southern Restored)

We selected eight natural Banksia woodland sites and two restored sites on the Swan Coastal Plain, in the Southwest of Western Australia. Banksia woodlands were listed in 2016 by the Australian government as an “Endangered Ecological Community” under the *EPBC Act* 1999. Less than 28% of the original woodland area remains around the Perth metropolitan area (Figure 1), and as such, these highly fragmented woodlands are a priority for ecological restoration (Ritchie et al., 2021). Natural Banksia woodland sites were selected as representatives of the landscape variability: two sites within a large remnant woodland (LR1 and LR2), four sites within an urban matrix (fragmented, FR1‐FR4), and two sites adjacent (AFR1 and AFR2) (within 200 m) to the two restored sites (RS1 and RS2) (Figure 1, Appendix S1). Sites were chosen to represent indicative reserve sizes, shapes, and internal characteristics of a reference system to capture the range of “naturalness” of what are potential reference sites for restoration evaluation. The experimental design is unbalanced, as at the time of study there were only two restored Banksia woodland sites that contained both *B. attenuata* and *B. menziesii* of reproductive age (of 14 and 15 years old) other than one within an active mining pit studied by Frick et al. (2014) for comparison to natural sites. We acknowledge that age and structural differences exist (Ritchie et al., 2017) (e.g., longevity up to 300 years old (Lamont et al., 2007)).

Metrics describing the properties of habitat remnants were calculated for each site: size (ha), mean proximity index (site isolation), total edge (m), density edge (m/ha), total edge contrast (percentage of boundary (m) with contrasting landscape), and edge contrast type (percentage of the boundary (m) with contrasting landscape of urban, floristic, or vegetation structure using R package *landscapemetrics*) (Hesselbarth et al., 2019) (Appendix S1). We assessed collinearity of these metrics and those that remained (with <0.7 similarity; site isolation, total edge, urban, floristic, and structure) were used for further analysis.

### 2.2 Study species

*Banksia attenuata* and *B. menziesii* are widespread Proteaceous trees or woody shrub species growing in deep sand (Collins et al., 2008). These dominant tree species of Banksia woodlands and shrublands (Kwongan) provide significant nectar or pollen resources to a large number of floral visitors (Ramsay, 1989; Ramsey, 1988a; Wooller & Wooller, 2001). Both *Banksia* species are obligately outcrossing (Scott, 1980) and dependent on animal‐mediated pollination by birds, mammals, or insects. *Banksia attenuata* flowers during the austral summer (November to February) (Wooller & Wooller, 2001) and has inflorescences that contain about 1,200–1,500 yellow sessile florets (up to 22 mm long) arranged orthogonally around a central woody axis, up to 5 cm wide and up to 5–26 cm long (Collins et al., 2008; Wooller et al., 1983). *Banksia menziesii* flowers during the austral autumn and winter (March to September), and has inflorescences that are up to 8 cm wide, 4–12 cm long, containing about 600–1,400 sessile florets (up to 71 mm long) ranging in color from pink to red (Collins et al., 2008; Ramsey, 1988b). It is likely the major source of nectar for honeyeaters during the winter months (Ramsay, 1989). Small marsupial honey possums (*Tarsipes rostratus*) also provide pollination services (Krauss et al., 2018; Wooller & Wooller, 2013); however, they are now mostly absent from urban Banksia woodlands and highly unlikely to naturally recolonize (How & Dell, 2000).

### 2.3 Floral resources and visitors

Floral attractiveness of sites was quantified during each floral visitor survey by counting the total number of inflorescences produced for 10 arbitrarily selected trees of each species in each study area. The study area was limited to 4–5 ha within each site due to differences in remnant size (detailed in Appendix S1). For each floral visitor survey, two spatially distant (>20 m) flowering trees with ≥5 inflorescences (labeled maternal) and their surrounding neighbors (noting their distance from the maternal tree) were monitored for floral visitors within the site, one by the same observer and the other by one volunteer. A list of bird species observed during the floral visitor surveys was collated to document differences in the general bird community (i.e., beyond nectarivores). *Banksia attenuata* was monitored in three summers (once in 2010/2011 and twice in each of 2011/2012 and 2012/2013) and *B. menziesii* was monitored over three winters (once 2011 and twice in each of 2012 and 2013), during days without rain or high wind. A total of 96 survey days were conducted in a consecutive sequence of 5‐day blocks during peak *Banksia* flowering for each *Banksia* species. On each survey day, visits to inflorescences by bird species were observed for eight, 10‐min census periods each hour, starting within 30 min of sunrise. Floral visitor abundance (a count of the number of visitors observed on a tree) was accepted as a proxy for effective pollination as previous studies have determined honeyeaters are the primary pollinators for both *Banksia* species (Ramsey, 1988a, 1988b; Scott, 1980; Whelan & Burbidge, 1980). Visitation was standardized as the number of foraging bouts to the number of inflorescences counted on the tree under observation. Visitation rate was calculated as the number of visits per inflorescence per 10 min of survey effort.

Observations of foraging behavior by floral visitors were made during survey periods based on point count observation method used by Ramsay (1989) and Whelan et al. (2009). Observers recorded the species and foraging behavior for each floral visit to a maternal tree as follows:

1. Visitation—foraging bouts were counted as the continuous tracking of a single floral visitor until lost from sight or survey time elapsed.
2. Intratree—the next inflorescence visited was located on the same tree.
3. Near neighbor—the next inflorescence visited was on an adjacent tree of *B. attenuata* or *B. menziesii* (<3 m).
4. Distant—the next inflorescence was not on a neighbor tree (<10 m).
5. To non‐*Banksia*—after the foraging bout the visitor visited another plant species.
6. Out of the site—after the initial foraging bout, the visitor moved out of the observation survey area (>10 m).
7. Probed inflorescence (yes/no)
8. Number of inflorescences visited and the time spent foraging—for each tree during a foraging bout.
9. Foraging interaction—during each foraging bout, any intraspecific or interspecific species displacement as recorded by species type and frequency.

We used general linear models (GLM) with a negative binomial to correct for overdispersion to test the effects of *Banksia* species, site types, and bird species (most common floral visitor) on floral visitor abundance. Linear models and model selection were conducted using R statistical environment version 3.6.1 (R Core Team, 2019) using packages *stats*, *lme4* (Bates et al., 2019), and *MASS* (Ripley et al., 2018). The final model structure used the backward selection function comparing the full model to smaller subsets based on Akaike's information criteria (AIC) and was implemented using the *StepAIC* function in the *MASS* package. Model significance (*P* values) were obtained using the Likelihood Ratio Tests (χ^2^), using the ANOVA function.

### 2.4 Diversity and composition of floral visitors

Diversity indices (number of species (*S*); species richness (Margalef's index *d* = (*S−*1)/ln *N*)) and Shannon–Wiener (*H*’) were used to compare floral visitors (visiting ≥1 inflorescence) between *Banksia* species (i.e., seasonal differences), among sites and among site types (large remnant, fragmented, adjacent, and restored). We assessed species‐abundance matrices using PRIMER v 6 (Clarke, 1993) to determine how bird species composition differed between seasons and among site types. Ordinations of species similarity were performed using nonmetric multidimensional scaling (NMDS) of Bray–Curtis distance. Analysis of Similarity (ANOSIM) was used to test for differences among site types in species composition, where a Global R value of <0.1 was inferred to indicate similarity (Clarke, 1993). Furthermore, a Similarity of Percentages (SIMPER) was used to identify which species were important in discriminating among site types.

### 2.5 Visitor behavior and foraging movements

We performed multiple generalized linear mixed‐effects models (GLMMs) with binomial error distribution to assess site and landscape metric effects on visitor probes (yes/no) to examine presence versus foraging. Survey number and the number of inflorescences on the maternal trees were set as random effects, and the data were analyzed by *Banksia* species. We used GLM with quasi‐Poisson error distribution to correct for overdispersion to test the effects of *Banksia* species, site types, and bird species on time spent foraging. Linear models and model selection were conducted using R statistical environment version 3.6.1 (R Core Team, 2019), using the exact method described under Floral Resources and Visitors.

Patterns of foraging movements were combined across surveys and seasons within each site and displayed as foraging movement network. These network graphs were created and measured for graph metrics using R package *igraph* (Csardi & Nepusz, 2006). The number of events and the directional movement after the first foraging bout on the focal *Banksia* tree under observation were displayed as graph edges. The number of *Banksia* inflorescences was averaged for each survey at each site and was represented as graph nodes to explore whether floral attraction was related to foraging movements. As these networks were a node‐level assessment differing in edge weight only, we utilized the methods of McDonald and Hobson (2018) to measure the distribution of observations within the network using *observed edge weight diversity* (*O*). Graph *strength* was used to make pairwise comparisons (ꭕ^2^) between each site network graph (see Delmas et al., 2019).

Pollinator Movement Index (PMI, Phillips et al., 2010) was used to estimate the distance bird pollinators traveled as a ratio between the distances traveled and distance to nearest forage tree from the observed tree. This provided an estimate of how far a pollinator moved to a tree with inflorescences compared with the minimum distance it could have moved to next to forage. Pollinators with a high PMI are presumed to move pollen further for a given density of trees because they move further than the minimum possible distance (Phillips et al., 2014). We compared PMI between site types (large remnant, fragmented, adjacent, and restored) and *Banksia* species using general linear models (GLM) with a Gaussian distribution and identity link function with data log(*x* + 1) transformed within the model to meet assumptions.

Displacement interactions during foraging bouts were combined across surveys and seasons within each site and displayed in an interaction network. As for foraging movement networks, interaction networks were created and measured for graph metrics using *igraph* (Csardi & Nepusz, 2006) and compared using *popgraph* packages (Dyer, 2015). The number and directionality of intraspecific and interspecific chases observed were displayed as edges in the graphs, and the proportional body size was represented as nodes in the graphs, to explore whether behavioral dominance was linked to body weight. Eigenvector centrality and density of these community‐level networks were measured using R package *igraph* (Csardi & Nepusz, 2006). These two measures give an indication of ecological complexity within the networks at each site (Delmas et al., 2019). Eigenvector centrality is a measure of the influence of a node in a network and a high eigenvector score means a node is connected to many nodes, which themselves are highly connected (Golbeck, 2015). Density of the network graph is the ratio of the number of edges and the number of possible edges (Golbeck, 2015). Structural congruence tests were used to make pairwise comparisons between each site's network graphs using *popgraph* (Dyer, 2015).

## 3 RESULTS

### 3.1 Floral resources and visitors

*Banksia* flowering within each season occurred simultaneously across all sites and at a similar intensity (*B. attenuata*: 8.2–11.4 and *B. menziesii*: 6.8–10.4 inflorescences per tree), except for the southern restored site (RS2; Appendix S2). *Banksia attenuata* trees in the southern restored site (RS2) produced significantly fewer inflorescences than the southern natural adjacent site (AFR2) (RS2 *n* = 4.1 ± 0.7; AFR2 *n* = 10.2 ± 1.0; *F*_9,270_ = 0.46, *p* = .02; Appendix S2). In contrast, *B. menziesii* trees in the same restored site (RS2) had significantly higher production of inflorescences than the natural adjacent site (RS2 *n* = 17.8 ± 0.5; AFR2 *n* = 8.8 ± 0.4; *F*_9,270_ = 3.04, *p* = .01; Appendix S2). Visitor abundance by *Banksia* species was significantly different (χ^2^ = 4.62, *n* = 200, *df* = 1, *p* < .05), as well as among the common visiting species (χ^2^ = 113.18, *n* = 200, *df* = 9, *p* < .001), however, there was no significant difference between site types (Appendix S3).

### 3.2 Visitor diversity and composition

A total of 1,878 observations of 21 bird species (nine floral visiting nectarivorous species and 12 non‐floral visiting species) were recorded for the entire study. Six species of honeyeaters, as well as the Silvereye (*Zosterops lateralis*), Australian Ringneck (*Barnardius zonarius* subsp. *semitorquatus*), and Rainbow Lorikeet (*Trichoglossus haematodus*; invasive species), were observed floral visitors. The most abundant nectarivores (honeyeaters) in decreasing order, across all sites, were the Brown Honeyeater (*Lichmera indistincta*), White‐Cheeked Honeyeater (*Phylidonyris niger*), Western Wattlebird (*Anthochaera lunulata*), New Holland Honeyeater (*Phylidonyris novae‐hollandiae*), Red Wattlebird (*Anthochaera carunculata*), and Western Spinebill (*Acanthorhynchus superciliosus*).

Total number of floral visiting species (*S*) was six in large remnants and restored sites and eight in fragmented and adjacent sites. The average number of bird species (*S ave*) observed foraging across all sites was significantly higher in winter than summer (winter *S* = 3.27 ± 0.25, summer *S = *2.5 ± 0.23; *F*_1_,_56_ = 4.38, *p* = .04). Fragmented sites on average had a lower number of species (FR1‐4: *S* = 2.08 ± 0.24) than all other sites (LR1‐2: *S* = 3.42 ± 0.34, AFR1‐2: *S* = 3.82 ± 0.36, RS1‐2: *S* = 3.27 ± 0.36, *F*_3,54_ = 7.05, *p* < .001). There was no significant difference in diversity metrics among sites (Margalef's species richness and Shannon–Wiener (Appendix S4).

Analysis of similarity (ANOSIM) showed no significant differences in pollinator assemblages between flowering seasons (summer and winter) (Global *R* = 0.04, *p* < .05); however, there was a significant difference in pollinator abundance (χ^2^ = 4.62, *df* = 1, *p* = .03, Appendix S5). The total composition and relative abundance of floral visitor species differed significantly between sites (ANOSIM Global *R* = 0.51, *p* = .001, Appendix S6). For example, Western Spinebills observed probing were at 1.4%–1.9% relative abundance in restored and fragmented sites, compared with 2.5%–6.1% in large and adjacent remnants (Appendix S6). Site composition was gathered into three groupings sharing 60% similarity, indicating that fragmented sites (FR1‐FR4) supported a different community composition to the northern (LR1, LR2, AFR1, and RS1) and southern (AFR2 and RS2) sites (Appendix S5).

The analysis of similarity (SIMPER) showed that adjacent (AFR1 and AFR2) and restored (RS1 and RS2) sites were the most similar (sharing <47% community composition), with fragmented sites the most dissimilar to large remnant and restored sites (sharing <20% community composition). The greatest species dissimilarity was among fragmented sites (sharing only 23% similarity), suggesting other individual site factors can influence differences in assemblages more so than shape or degree of isolation of these fragments.

### 3.3 Visitor behavior

**FIGURE 2:**
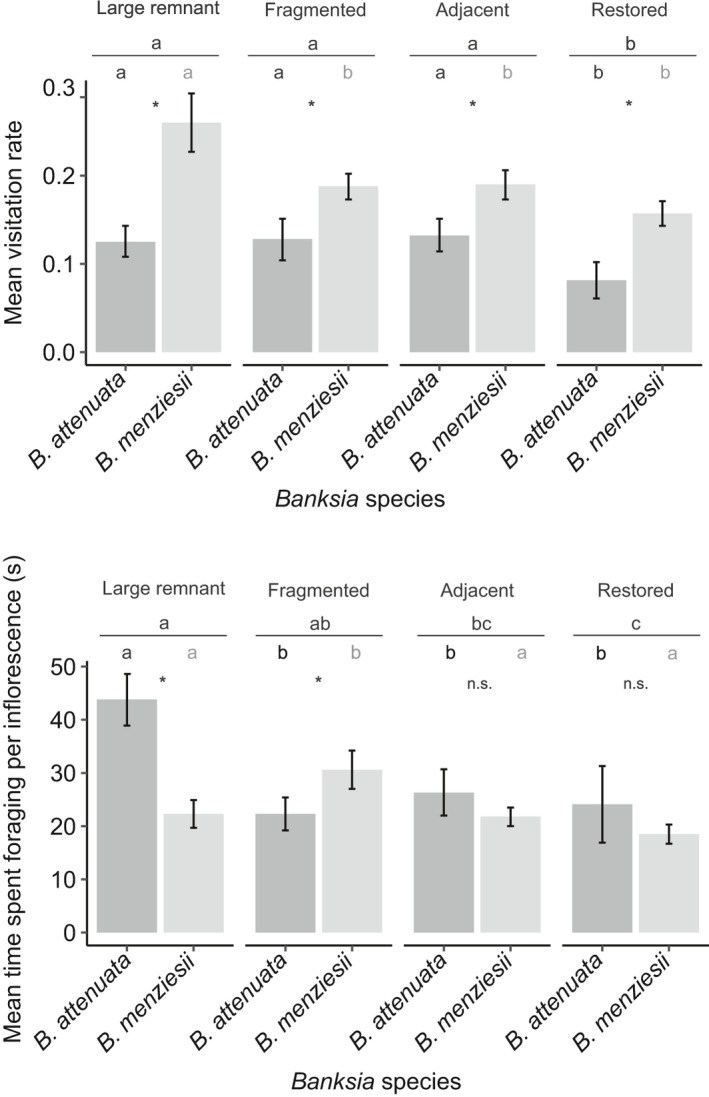
Pollinator behavior for all birds visiting *Banksia attenuata* and *B. menziesii* across site types. (a) Mean visitation rate (number of visits per inflorescence per 10 min) and (b) time spent foraging per inflorescence (seconds). Letters above the line indicate significant differences between site type and the letters below the line indicate significant differences between site types separated by *Banksia* species, with corresponding letters above each bar; *B. attenuata* left; *B*. *menziesii* right. Error bars indicate standard errors and asterisks indicate significant differences between *Banksia* species within site type (alpha = 0.05, generated from generalized linear models Tables Appendix S6)

A total of 1,173 foraging bouts were recorded, with foraging activity and visitation being higher at all sites for autumn/winter flowering *B. menziesii* (*n* = 1,134 visits, 81.1% of inflorescences probed, Appendix S7) than the summer flowering *B. attenuata* (*n* = 761 visits, Wald χ^2^ = 16.89, *df* = 1, *n* = 2,160, *p* < .001; 67.9% probed, Wald χ^2^ = 32.66, *df* = 1, *n* = 2,160, *p* < .001) (Figure 2a and Appendix S3 and S7). A total of 245 foraging bouts were observed within the natural large remnants, 311 within fragmented remnants, 358 within natural adjacent sites, and 259 within restored sites.

Landscape metrics of edge contrasts for urban (Wald χ^2^ = 5.59, *df* = 1, *p* = .02) and structure (χ^2^ = 13.44, *df* = 1, *p* < .001) factors were the only ones detected as significantly influencing *B. menziesii* floral probing (Appendix S3). The urban (χ^2^ = 5.35, *df* = 1, *p* = .02), floristic (χ^2^ = 13.99, *df* = 1, *p* < .001), and structure (χ^2^ = 9.22, *df* = 1, *p* = .002) factors of edge contrasts, and site isolation (χ^2^ = 4.75, *df* = 1, *p* = .03) metrics significantly influenced floral probing in *B. attenuata* (Appendix S3). Landscape metrics indicated increased site edge contrasts were common features of fragmented remnants and restored sites (Appendix S1). Visitation rates were higher for *B*. *menziesii*, although time spent foraging was significantly lower in comparison to *B. attenuata* (Figure 2a and b). Visitation (Figure 2a) and the average time spent foraging per inflorescence (Figure 2b) were significantly lower within restored sites (RS = 19.4 s ± 1.9) than large remnants (LR = 34.2s ± 3.1) or fragmented remnants (FR = 28.4s ± 2.8) (χ^2^ = 57.34, *n* = 1651, *df* = 3, *p* < .001) (Figure 2b, Appendix S3). The foraging time spent within adjacent fragments (AFR = 23.2s ± 1.8) was not significantly different from fragmented or restored sites and however was significantly different from large remnants (χ^2^ = 57.34, *n* = 1651, *df* = 3, *p* < .001) (Figure 2b, Appendix S3).

### 3.4 Foraging movements

**FIGURE 3:**
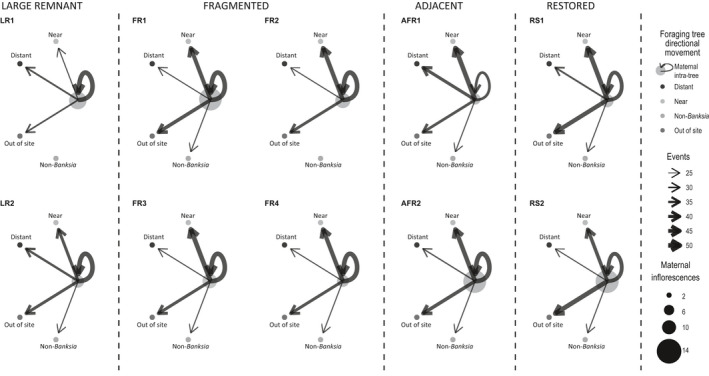
Foraging movement network graphs (node‐level assessment): Bird movements per site on *Banksia attenuata* and *B. menziesii* visualized as foraging movement network graphs. Events are total observations of movement after the initial probe foraging event. Nodes represent the location traveled after the initial foraging bout on the maternal tree; maternal tree node size indicates the average number of inflorescences on the observed tree; arrows indicate the directional movement after the first foraging bout and arrow width (i.e., edge weight) indicates the number of events observed

**FIGURE 4:**
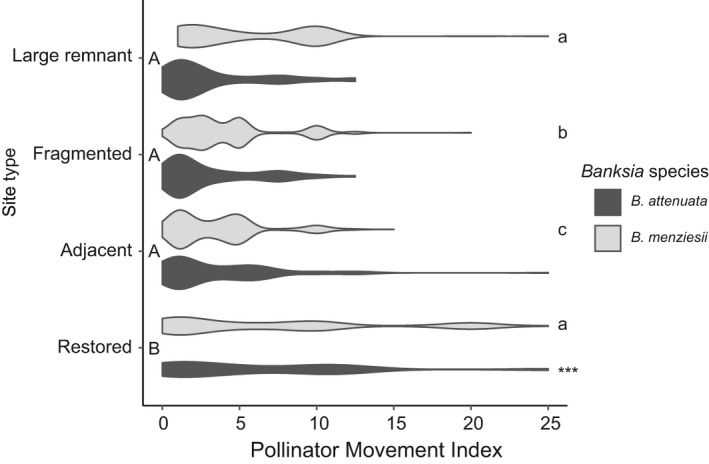
Violin plot showing the Pollinator Movement Index (PMI) for foraging behavior between site type (large remnant, fragmented, adjacent, and restored) for *Banksia attenuata* and *B. menziesii* flowering. Uppercase letters to the left of the figure near the y‐axis indicate significant differences (*p* ≤ .05) between site types. The lowercase letters on the right‐hand side indicate significant differences for *B. menziesii* PMI. The asterisk on the right‐hand side indicates significant differences for *B. attenuata* PMI. Note: No observations were made of zero PMI for *B. menziesii* within large remnants

Intratree foraging was the most frequent movement by birds at all sites for both *Banksia* species (Figure 3). Restored site two (RS2) had the lowest observed edge weight diversity (*O* = 0.68) whereas RS1 had the highest (*O* = 0.76) (Table S8). The most common subsequent movement to this was to near neighbor trees, except for LR1 and RS2 in which the majority of birds flew out of the site (24% and 38%, respectively; Figures 3 and 4, Appendix S8 and S9, Table S9). Foraging on *B. attenuata* occurred at significantly smaller distances (<5 m) within large, fragmented, and adjacent remnants in comparison with restored sites (>10 m) (*p* < .05, Figure 4). Foraging on *B. menziesii* occurred at much larger distances (up to 25 m) with the pattern of foraging between large remnant and restored sites being equivalent. Long distance travel (out of site) after foraging bouts occurred more frequently in the restored sites (Figure 3) and therefore generated a significantly different PMI between restored sites and all others (Figure 4).

**FIGURE 5:**
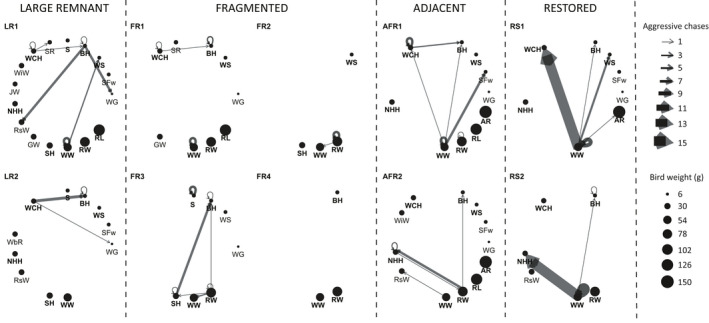
Interaction network graphs (community‐level assessment): The diversity of bird species at all sites and the displacement interactions observed for both *Banksia* species. Circle size indicates proportional body weight (g) (sourced from Ford, 1979; McFarland, 1986; Newland & Wooller, 1985). Circular arrows indicate intraspecific chases and arrows between bird species indicate direction of interspecific chases. Bird names in bold text are nectarivores; S, Silvereye; BH, Brown Honeyeater; WS, Western Spinebill; SFw, Splendid Fairy‐wren; WG, Western Gerygone; AR, Australian Ringneck; RL, Rainbow Lorikeet; RW, Red Wattlebird; WW, Western Wattlebird; SH, Singing Honeyeater; GW, Australian Golden Whistler; RsW, Rufous Whistler; NHH, New Holland Honeyeater; JW, Jacky Winter; WiW, Willie Wagtail; WCH, White‐cheeked Honeyeater; SR, Scarlet Robin

Intraspecies and interspecies displacement interactions (interrupted foraging) and aggressive chases by larger‐bodied species were higher in frequency within restored sites compared with all others (RS1 *n* = 24, RS2 *n* = 21; eigenvector centrality RS1 = 16.00, RS2 = 20.21, range of other sites 0–6.73; Figure 4). Aggressive chases and displacement of foraging honeyeaters were observed at all sites, except one fragmented site (FR4; Figure 5), with Western Wattlebirds being the main aggressor. The greatest number of interactions (network edges) between multiple species (network nodes) was observed within large remnant and adjacent sites (Figure 5). Congruency between bird interaction networks was found between large and fragmented remnants (*p* = .004) and fragmented and adjacent remnants (*p* = .001) (Appendix S10). Overall, a greater proportion of movements to distant trees were recorded for Brown, White‐cheeked, and New Holland Honeyeaters, in part due to their displacement by larger‐bodied honeyeaters (i.e., Red Wattlebirds or Western Wattlebirds; Figure 5).

## 4 DISCUSSION

An understanding of the temporal and spatial variability of plant–pollinator mutualisms in natural sites is critical for the creation of completion criteria and the assessment of restored sites (Burkle & Alarcon, 2011; Forup et al., 2008; Moreno‐Mateos et al., 2020). The community of *Banksia* bird visitors varied in composition, richness, foraging movement distances, and aggression among sites in this study. Differences in the composition were associated with the size and degree of fragmentation in natural sites, and this did not differ between seasons. The smaller fragmented sites, on average, had lower species richness of honeyeaters than large remnant sites, a trend common to fragmented landscapes (Davis & Wilcox, 2013; Ford et al., 2009; Marzluff & Ewing, 2001). Encouragingly, restored sites within this fragmented landscape had similar bird species composition to their adjacent natural remnants; however, bird foraging behavior varied. More frequent aggressive chases were observed in restored sites, resulting in more movements out of the surveyed areas, than observed in natural sites. The variability observed in the composition and behavior of bird pollinators for Banksias in the natural reference sites created a broad completion target against which restored sites were assessed. Ultimately, these restored sites met this target and achieved the restoration objective of functional pollinator services, albeit with clear differences.

Banksia flowers in this study were visited by as many as seven common honeyeater species. The reproductive outcome for each visitor depends on pollinator effectiveness associated with foraging in combination with plant resource allocation (Ramsay, 1989). The community composition and foraging behavior of bird‐pollinator species are influenced by their sensitivity to landscape disturbance and response to interspecies interactions (territoriality) (Armstrong, 1991; Ford & Paton, 1982; McFarland, 1986; Phillips et al., 2014; Ramsay, 1989; Ramsey, 1988b). Site isolation, differences in vegetation structure, and urbanization of the surrounding landscape influenced foraging in this study and are likely the causes of the dissimilarity in species composition among fragmented remnants (Clergeau et al., 2001; Munro et al., 2011) and reduced community network size (Tylianakis & Morris, 2017). These results are common with local bird community observations, with 50% lower species richness in suburban gardens than adjacent Banksia woodland (Davis & Wilcox, 2013).

The absence or rarity of small‐bodied Western Spinebills within fragmented and restored sites suggests that the species may be sensitive to disturbance. Davis and Wilcox (2013) noted the sensitivity of Western Spinebills and also reported the impact of barriers to movement, like roads (see Johnson et al., 2017). The absence of Western Spinebills within restored Banksia woodlands was also observed by Comer and Wooller (2002), who suggested that it was not the lack of vegetation cover that prevented visitation, but possibly aggression by larger honeyeaters. Aggression was observed at higher frequency within restored sites of our study, reducing honeyeater foraging on Banksias.

Increased aggression by honeyeaters is associated with increased floral resources (Armstrong, 1991; Ford, 1979; Phillips et al., 2014). Winter flowering of *B. menziesii* in restored sites was greater than in adjacent natural sites. Large‐bodied Western Wattlebirds generally established territories and dominated the abundant nectar resources within restored sites, forcing smaller‐bodied birds (e.g., Western Spinebills, Brown Honeyeaters) out of these sites. Newland and Wooller (1985) observed that Western Wattlebirds dominated natural Banksia woodland sites when flowering density was high, while smaller resident honeyeaters exploited dispersed floral resources throughout the year.

Globally, other nectivorous species display similar patterns of aggression in response to changes in flower density and/or nectar availability (Carpenter, 1987; Franceschinelli & Bawa, 2000; López‐Segoviano et al., 2018; Smith‐Ramirez & Armesto, 2003). The likely reason behind this aggression and observed dominance hierarchy is based on the energy requirements of different sized birds (Armstrong, 1991; Mac Nally & Timewell, 2005). Energy intake rates increase with body size. For example, larger‐bodied honeyeaters (e.g., Western Wattlebirds) require a higher intake of nectar than smaller‐bodied honeyeaters (e.g., New Holland Honeyeaters), and both require higher intake rates than the even smaller‐bodied Eastern Spinebills (Mitchell & Paton, 1990).

Floral resource availability is considered one of the major determining factors of honeyeater presence (Ford, 1979; Ford & Paton, 1982). However, we found that the differing flowering intensity between restored and adjacent sites was not correlated with visitation within these sites or at all other sites. Low honeyeater visitation during summer within restored sites (particularly at RS2) may be attributed to reduced floral attraction because of lower *B. attenuata* floral abundance, and the overall lower floristic diversity, in comparison with the adjacent and surrounding natural remnants (Ritchie et al., 2017).

Site isolation was a significant factor influencing foraging during summer, likely due to the greater energetic expenditure required to traverse fragmented landscapes (Tomlinson et al., 2014). Maximizing foraging efficiency may therefore explain the increased time spent on *B. attenuata* inflorescences during summer (McCallum et al., 2013) in comparison with winter flowering *B. menziesii,* although overall visitation was lower than *B. menziesii*. A greater abundance of co‐flowering plant species and associated invertebrates (additional food sources) are known to occur in Banksia woodlands during summer than in winter months (Whelan & Burbidge, 1980). The energetic requirements of birds are also associated with ambient temperature and their need to consume energy sources when they are available, such as invertebrates, when they are known to occur in greater abundance during warm periods (Timewell & Mac Nally, 2004; Whelan & Burbidge, 1980). Foraging behavior, and therefore pollen dispersal distances, may change to accommodate these energy requirements in these altered landscapes. This behavior highlights the importance of considering the local site and the wider landscape in conjunction with seasonality (i.e., overlapping flowering phenology) when implementing restoration design.

Differences in bird foraging movements can influence the pollination service they provide to plants, which can impact the comparative seed production between sites (Ritchie et al., 2019). We found bird–pollinator movements to be largely (35%–47%) within trees or between near neighbors in all sites, consistent with previous studies (Ramsay, 1989; Vaughton, 1990). Bird movements between more distant plants became more common as remnant size increased, a trend also observed in other fragmented landscapes with bird‐pollinated plants (Llorens et al., 2012; Yates et al., 2007). There are thus likely different genetic consequences for natural and restored populations of *Banksia* because of known differences in spatial population genetic structure, and inbreeding avoidance mechanisms (see Krauss et al., 2009; Ritchie et al., 2017, 2019; Ritchie & Krauss, 2012), which ultimately determine reproductive success (Wooller & Wooller, 2001). The observed differences in visitation and movement patterns among sites reflect the natural variability of interactions and resource availability in natural and disturbed parts of this urban‐dominated landscape. This emphasizes the range of plant–pollinator mutualisms for restored Banksias; however, the challenges of restoring a diverse bird–pollinator community remain (Pauw, 2019).

Observational studies of pollinator foraging movements in response to nectar resource availability, spatial arrangement of resources, and the interactions among pollinator species have practical implications for restoration planting design (Comer & Wooller, 2002; McCallum et al., 2018). For example, territorial bird pollinators of *Embothrium coccineum* (Proteaceae) in Chile were largely restricted to defending clumps of 3–5 adjacent flowering trees, with more diverse pollinator assemblages visiting undefended pasture trees (Smith‐Ramirez & Armesto, 2003). Canopy cover, tree species, and patch size within restored sites have also been observed to influence bird visitation (Fink et al., 2009). Planting design strategies should consider community dynamics, look to attract and establish a greater diversity of pollinator species, and integrate key resources needed to establish “pollinator‐friendly” environments (Dixon, 2009). These should include addressing the requirements for invertebrate recolonization within restored sites (e.g., woody debris, see Lythe et al. (2017)), as they are protein sources for birds.

The morphological (e.g., large‐bodied) and behavioral traits of these generalist bird pollinators facilitates their access to more distant resources, supporting their existence in fragmented landscapes (Hagen et al., 2012; Yates et al., 2007). These traits decrease the risk of failure in the delivery of pollinator services (Wessinger, 2020). However, as habitat loss intensifies, particularly in urban ecosystems, the distance between remnants increases, reducing bird mobility because of their high resource requirements (Hagen et al., 2012). Knowledge of which pollinators are negatively impacted by habitat fragmentation and urbanization is required to develop an understanding of the impacts (e.g., for pollinator services) and solutions for their reinstatement. Consideration needs to be given to the landscape positioning and connectivity of restored sites to ensure the movement of pollinators’ communities and maintenance of pollinator services between these remnants (see Ritchie et al., 2017, 2019).

The results for these *Banksia* species support the suggestion that pollinator interactions and network structure are often inherently plastic (Burkle & Alarcon, 2011). Thus, for many plants the exact identity of their generalist pollinator community may be less important than having a diverse mixture within the functional group (Hagen et al., 2012). The conservation and restoration of urban ecosystems require study of the temporal and spatial variation of pollinator communities and their interactions in order to quantify the return of ecosystem functionality in restored sites. Doing so will guide restoration strategies for planting design beyond solely re‐establishment and support conservation and land management efforts for the long‐term restoration of ecosystem complexity and population self‐sustainability.

## AUTHOR CONTRIBUTIONS

**Alison L. Ritchie:** Conceptualization (equal); Data curation (lead); Formal analysis (lead); Investigation (lead); Methodology (lead); Validation (equal); Visualization (equal); Writing‐original draft (lead); Writing‐review & editing (equal). **Carole P. Elliott:** Formal analysis (equal); Investigation (equal); Methodology (equal); Supervision (equal); Validation (equal); Writing‐review & editing (equal). **Elizabeth A. Sinclair:** Conceptualization (equal); Investigation (equal); Project administration (equal); Supervision (equal); Validation (equal); Writing‐review & editing (equal). **Siegfried L. Krauss:** Conceptualization (equal); Funding acquisition (lead); Investigation (equal); Methodology (equal); Project administration (lead); Resources (lead); Supervision (equal); Validation (equal); Writing‐review & editing (equal).

## Supporting information

Appendix S1

Appendix S2

Appendix S3

Appendix S4

Appendix S5

Appendix S6

Appendix S7

Appendix S8

Appendix S9

Appendix S10

## Data Availability

Data are available from the Dryad Digital Repository https://doi.org/10.5061/dryad.ncjsxksvk.
