# Supplementary material for: Restored and remnant Banksia woodlands elicit different foraging behavior in avian pollinators
Source: Ecol Evol. 2021 Jul 27;11(17):11774–85. doi: 10.1002/ece3.7946 (PMC8427588; doi:10.1002/ece3.7946)
Supplement: Supplementary file 1 — Appendix S1 [file ECE3-11-11774-s008.docx]

Appendix S1. Site location and characteristics.

| Site, Latitude, longitude | **Neaves North** | **Neaves South** | **Hepburn Park** | **Highview Park** | **Marangaroo Conservation Reserve** | **Paloma Park** | **Northern adjacent** | **Southern adjacent** | **Northern restored** | **Southern restored** |
| --- | --- | --- | --- | --- | --- | --- | --- | --- | --- | --- |
|  | 31 40’28”S, 115 53’44”E | 31 40’09”S, 115 53’49”E | 31 49’09”S, 115 52’29”E | 31 49’38”S, 115 51’31”E | 31 49’44”S, 115 50’09”E | 31 49’57”S, 115 51’06”E | 31 47’07”S, 115 56’23”E | 32 06’18”S, 115 52’07”E | 31 47’09”S, 115 56’32”E | 32 06’28”S, 115 52’01”E |
| **Metrics** | Non-fragmented | | Fragmented | | | | | | Restored | |
| Code | LR1 | LR2 | FR1 | FR2 | FR3 | FR4 | AFR1 | AFR2 | RS1 | RS2 |
| Type | Large remnant | Large remnant | Fragmented | Fragmented | Fragmented | Fragmented | Adjacent | Adjacent | Restored | Restored |
| Size (ha) | >750 | >750 | 9.8 | 10.9 | 22.4 | 5.1 | 18.4 | 14 | 33.2 | 25.6 |
| Area surveyed (ha) | 4.7 | 5 | 4.2 | 4.9 | 4.7 | 5.1 | 4.7 | 4.1 | 4.9 | 4.1 |
| Isolation (km)^Ϯ^ | 0 | 0 | 1.28 | 1.28 | 1.87 | 1.46 | 1.7 | 1.25 | 1.27 | 0.85 |
| Total edge (km) | 12.34 | 12.55 | 1.3 | 1.33 | 2.93 | 0.93 | 2.04 | 2.03 | 2 | 2.17 |
| Density edge (m/ha) | 16.45 | 16.73 | 132.86 | 121.74 | 130.88 | 183.99 | 110.98 | 144.93 | 60.36 | 84.92 |
| Edge contrast** | 4.05 | 2.41 | 48.71 | 48.72 | 52.73 | 64.96 | 11.01 | 27.43 | 26.4 | 20.7 |
| Urban* | 0 | 0 | 0.6 | 0.6 | 0.58 | 0.83 | 0 | 0.28 | 0.15 | 0.15 |
| Floristic* | 0.27 | 0.27 | 0.06 | 0.15 | 0.38 | 0.11 | 0.48 | 0.25 | 0.75 | 0.05 |
| Structure* | 0 | 0.09 | 0.28 | 0.15 | 0.38 | 0.11 | 0.38 | 0.26 | 0.36 | 0.85 |

^Ϯ^ Mean proximity index, uses the average distance between the focal site and each of the other remnant patches within a 2km search radius.

**Proportion of maximum contrast along the perimeter edge of the focal site remnant (i.e. difference from Banksia woodland). Note that this index is a relative measure.

*Proportion of maximum contrast along the perimeter edge of the focal site remnant for urban (i.e. roads, infrastructure, housing), floristics (vegetation not Banksia woodlands i.e. pine forest), and structure (change in floristic structure, i.e. change between restored and remnant, intact or disturbed Banksia woodlands). Note that this index is a relative measure. A description of these metrics is available from <http://www.umass.edu/landeco/teaching/landscape_ecology/schedule/chapter9_metrics.pdf>

(accessed May 2015).


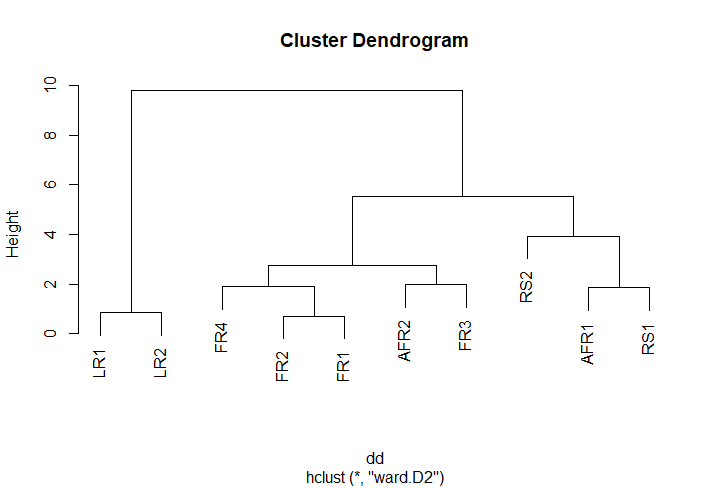


**Figure S1.** Cluster dendrogram based on Bray-Curtis dissimilarity of site landscape characteristics from Appendix S1, created using R statistical environment version 3.6.1 (R Core Team, 2019) using packages *vegan* (Oksanen et al., 2013).

OKSANEN, J., BLANCHET, F. G., KINDT, R., LEGENDRE, P., MINCHIN, P. R., O’HARA, R., SIMPSON, G. L., SOLYMOS, P., STEVENS, M. H. H. & WAGNER, H. 2013. Package ‘vegan’. *Community ecology package, version,* 2**,** 1-295.

R CORE TEAM 2019. R: A Language and Environment for Statistical Computing. Vienna, Austria: R Foundation for Statistical Computing.
