# Supplementary material for: Restored and remnant Banksia woodlands elicit different foraging behavior in avian pollinators
Source: Ecol Evol. 2021 Jul 27;11(17):11774–85. doi: 10.1002/ece3.7946 (PMC8427588; doi:10.1002/ece3.7946)
Supplement: Supplementary file 2 — Appendix S2 [file ECE3-11-11774-s005.docx]

**Appendix S2.
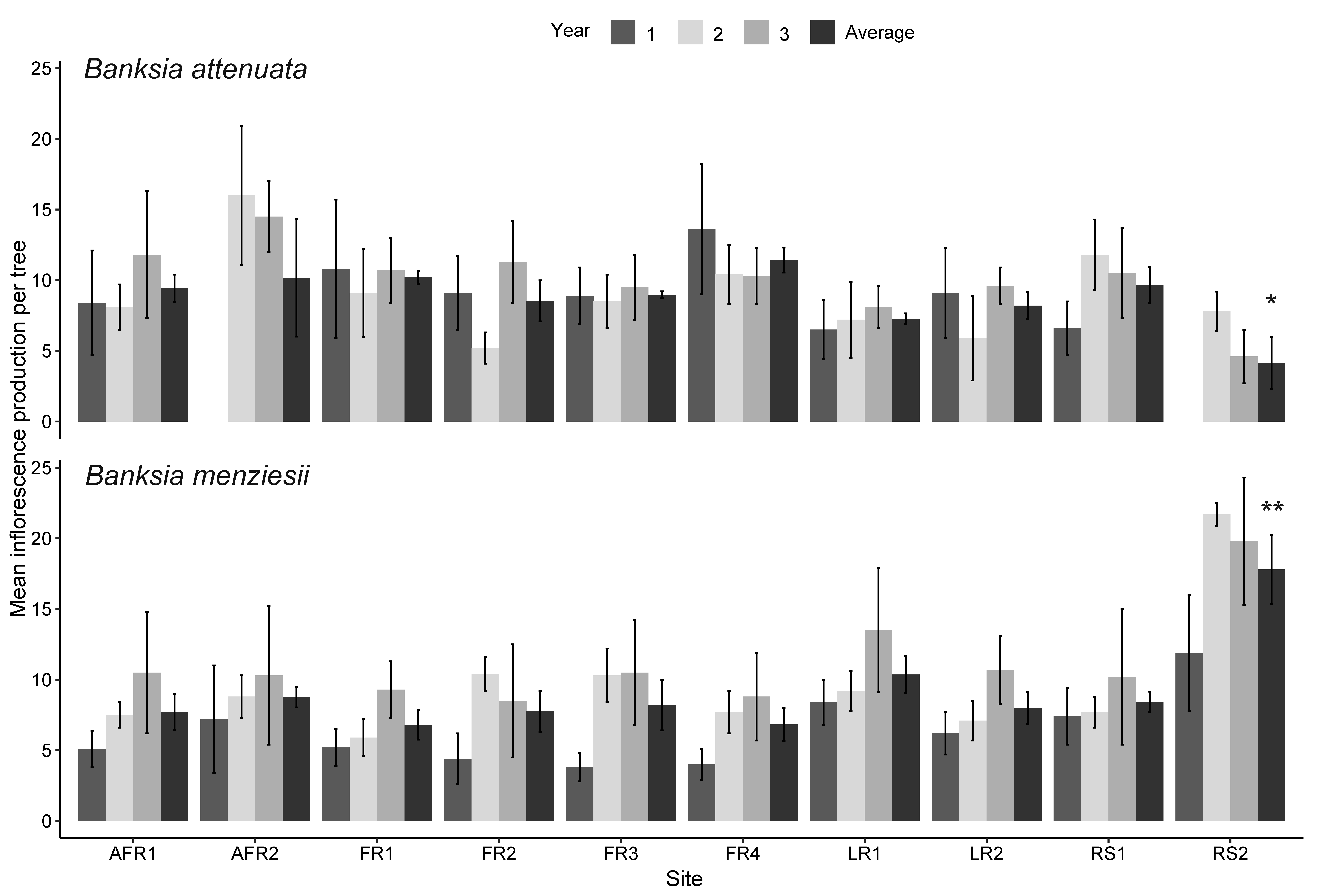
Figure** Mean inflorescence count per tree for 10 trees in each site for *Banksia attenuata* (summer flowering) and *B. menziesii* (winter flowering). No results were recorded for *B. attenuata* flowering in AFR2 and RS2 for summer (Year 1)**.** Significance denoted by alpha ‘*’ 0.05, ‘**’ 0.01.
