# Supplementary material for: Restored and remnant Banksia woodlands elicit different foraging behavior in avian pollinators
Source: Ecol Evol. 2021 Jul 27;11(17):11774–85. doi: 10.1002/ece3.7946 (PMC8427588; doi:10.1002/ece3.7946)
Supplement: Supplementary file 3 — Appendix S3 [file ECE3-11-11774-s003.docx]

**Appendix S3.** Results from the best fitting generalized linear models for bird visits (abundance) and general linear mixed models for probing, visitation and time foraging on *Banksia menziesii* and *B. attenuata* inflorescences. Model fit assessed with the Akike’s Information Criterion (AIC).

| Response | Model | ꭕ^2^, *P* | df | Predictors (fixed effect) | N | Estimate | ±SE | *Z* | *P* | R^2^ | BIC | AIC |
| --- | --- | --- | --- | --- | --- | --- | --- | --- | --- | --- | --- | --- |
| **Bird visits (abundance)** | ***Banksia* species** | 4.62* | 1 | Intercept | 200 | 1.64 | 0.26 | 6.40 | 0.00 | 0.02 | 964.56 | 954.66 |
|  |  |  |  | *B. menziesii* |  | 0.79 | 0.36 | 2.17 | 0.03 |  |  |  |
|  |  |  |  |  |  |  |  |  |  |  |  |  |
| **Bird visits (abundance)** | **Site type** | 4.04 | 3 | Intercept | 200 | 2.58 | 0.40 | 6.41 | 0.00 | 0.02 | 975.71 | 959.22 |
|  |  |  |  | Fragmented |  | -0.94 | 0.49 | -1.91 | 0.06 |  |  |  |
|  |  |  |  | Natural |  | -0.46 | 0.57 | -0.80 | 0.42 |  |  |  |
|  |  |  |  | Restored |  | -0.34 | 0.57 | -0.60 | 0.55 |  |  |  |
|  |  |  |  |  |  |  |  |  |  |  |  |  |
| **Bird visits (abundance)** | **Bird species** | 113.18*** | 9 | Intercept | 200 | -1.90 | 0.71 | -2.68 | 0.01 | 0.34 | 929.94 | 893.66 |
|  |  |  |  | Brown Honeyeater |  | 4.86 | 0.82 | 5.92 | 0.00 |  |  |  |
|  |  |  |  | New Holland Honeyeater |  | 4.16 | 0.82 | 5.05 | 0.00 |  |  |  |
|  |  |  |  | Rainbow Lorikeet |  | 0.51 | 0.93 | 0.55 | 0.58 |  |  |  |
|  |  |  |  | Red Wattlebird |  | 3.74 | 0.82 | 4.53 | 0.00 |  |  |  |
|  |  |  |  | Silvereye |  | 1.79 | 0.85 | 2.10 | 0.04 |  |  |  |
|  |  |  |  | Singing Honeyeater |  | 1.10 | 0.88 | 1.24 | 0.21 |  |  |  |
|  |  |  |  | White-Cheeked Honeyeater |  | 5.02 | 0.82 | 6.12 | 0.00 |  |  |  |
|  |  |  |  | Western Spinebill |  | 3.01 | 0.83 | 3.63 | 0.00 |  |  |  |
|  |  |  |  | Western Wattlebird |  | 4.88 | 0.82 | 5.94 | 0.00 |  |  |  |
|  |  |  |  |  |  |  |  |  |  |  |  |  |
| **Probed (y/n)** | **Site type** | 12.46** | 3 | Intercept | 1399 | 1.56 | 0.28 | 5.58 | 0.00 | 0.19 | 1343.11 | 1311.65 |
| ***Banksia menziesii*** | Random effects: |  |  | Fragmented |  | 0.17 | 0.23 | 0.75 | 0.45 |  |  |  |
|  | Survey |  |  | Natural |  | 0.04 | 0.30 | 0.12 | 0.90 |  |  |  |
|  | Infl. produced |  |  | Restored |  | -0.54 | 0.21 | -2.62 | 0.01 |  |  |  |
|  |  |  |  |  |  |  |  |  |  |  |  |  |
|  | **Landscape** |  |  | Intercept | 1399 | 1.69 | 0.29 | 5.89 | 0.00 | 0.19 | 1326.61 | 1300.40 |
|  | Random effects: | 5.59* |  | Urban |  | -1.42 | 0.39 | -3.66 | 0.00 |  |  |  |
|  | Survey | 13.43*** |  | Structure |  | 0.92 | 0.39 | 2.36 | 0.02 |  |  |  |
|  | Infl. produced |  |  |  |  |  |  |  |  |  |  |  |
|  |  |  |  |  |  |  |  |  |  |  |  |  |
| **Inflorescences visited** | **Site type** |  |  | Intercept | 1134 | -1.62 | 0.14 | -11.31 | 0.00 | 0.08 | 3015.16 | 2984.96 |
| ***Banksia menziesii*** | Random effects: | 13.80** |  | Fragmented |  | -0.00 | 0.08 | -0.02 | 0.99 |  |  |  |
|  | Survey |  |  | Natural |  | 0.05 | 0.10 | 0.54 | 0.59 |  |  |  |
|  | Infl. produced |  |  | Restored |  | -0.22 | 0.08 | -2.89 | 0.00 |  |  |  |
|  |  |  |  |  |  |  |  |  |  |  |  |  |
| **Probed (y/n)** | **Site type** | 11.45** |  | Intercept | 761 | 1.36 | 0.39 | 3.45 | 0.00 | 0.31 | 886.10 | 858.29 |
| ***Banksia attenuata*** | Random effects: |  |  | Fragmented |  | -0.11 | 0.36 | -0.32 | 0.75 |  |  |  |
|  | Survey |  |  | Natural |  | -0.41 | 0.36 | -1.13 | 0.26 |  |  |  |
|  | Infl. produced |  |  | Restored |  | -1.48 | 0.47 | -3.18 | 0.00 |  |  |  |
|  |  |  |  |  |  |  |  |  |  |  |  |  |
|  | **Landscape** |  |  | Intercept | 761 | 6.38 | 3.02 | 2.11 | 0.03 | 0.32 | 888.93 | 851.86 |
|  | Random effects: | 5.35* |  | Urban |  | -1.96 | 0.85 | -2.31 | 0.02 |  |  |  |
|  | Survey | 13.98*** |  | Floristic |  | -3.98 | 1.07 | -3.74 | 0.00 |  |  |  |
|  | Infl. produced | 9.21** |  | Structure |  | -3.04 | 1.00 | -3.04 | 0.00 |  |  |  |
|  |  | 4.75* |  | Isolation |  | 1.15 | 0.53 | 2.18 | 0.03 |  |  |  |
|  |  | 2.32 |  | Total edge |  | -0.50 | 0.33 | -1.52 | 0.13 |  |  |  |
|  |  |  |  |  |  |  |  |  |  |  |  |  |
| **Inflorescences visited** | **Site type** | 1.56 |  | Intercept | 517 | -1.92 | 0.16 | -12.09 | 0.00 | 0.07 | 1481.30 | 1455.81 |
| ***Banksia attenuata*** | Random effects: |  |  | Fragmented |  | 0.03 | 0.15 | 0.18 | 0.86 |  |  |  |
|  | Survey |  |  | Natural |  | -0.08 | 0.14 | -0.53 | 0.59 |  |  |  |
|  | Infl. produced |  |  | Restored |  | -0.18 | 0.20 | -0.93 | 0.35 |  |  |  |
|  |  |  |  |  |  |  |  |  |  |  |  |  |
| **Time foraging** | ***Banksia* species** | 18.75*** |  | Intercept | 1651 | 3.15 | 0.03 | 95.93 | 0.00 | 0.02 | 3740.60 | 3724.37 |
| Log(time/visited) |  |  |  | *B. menziesii* |  | -0.23 | 0.04 | -5.80 | 0.00 |  |  |  |
|  | **Site type** | 57.34*** |  | Intercept | 1651 | 2.91 | 0.03 | 91.44 | 0.00 | 0.07 | 3684.61 | 3657.56 |
|  |  |  |  | Fragmented |  | 0.19 | 0.05 | 3.87 | 0.00 |  |  |  |
|  |  |  |  | Natural |  | 0.37 | 0.05 | 7.22 | 0.00 |  |  |  |
|  |  |  |  | Restored |  | -0.16 | 0.05 | -3.23 | 0.00 |  |  |  |
|  |  |  |  |  |  |  |  |  |  |  |  |  |
| **Time foraging** | **Site type** | 30.36*** |  | Intercept | 1134 | 2.89 | 0.04 | 79.80 | 0.00 | 0.06 | 2418.62 | 2393.45 |
| ***Banksia menziesii*** |  |  |  | Fragmented |  | 0.26 | 0.05 | 4.81 | 0.00 |  |  |  |
| Log time Gaussian glm |  |  |  | Natural |  | 0.06 | 0.07 | 0.91 | 0.36 |  |  |  |
|  |  |  |  | Restored |  | -0.18 | 0.05 | -3.40 | 0.00 |  |  |  |
|  |  |  |  |  |  |  |  |  |  |  |  |  |
| **Time foraging** | **Site type** | 43.09*** |  | Intercept | 517 | 2.94 | 0.06 | 48.89 | 0.00 | 0.14 | 1221.36 | 1200.12 |
| ***Banksia attenuata*** |  |  |  | Fragmented |  | -0.01 | 0.10 | -0.14 | 0.89 |  |  |  |
| Log time Gaussian glm |  |  |  | Natural |  | 0.60 | 0.08 | 7.22 | 0.00 |  |  |  |
|  |  |  |  | Restored |  | -0.01 | 0.11 | -0.09 | 0.93 |  |  |  |

*P* = * 0.05; ** 0.01; ***0.001
