# Supplementary material for: Restored and remnant Banksia woodlands elicit different foraging behavior in avian pollinators
Source: Ecol Evol. 2021 Jul 27;11(17):11774–85. doi: 10.1002/ece3.7946 (PMC8427588; doi:10.1002/ece3.7946)
Supplement: Supplementary file 4 — Appendix S4 [file ECE3-11-11774-s002.docx]

**Appendix S4.** Diversity metrics of observed bird species visitation total at each site and during each season. Total species richness (*S*), Average species richness (*S ave*), Margalef’s diversity index (*d*), and Shannon-Weiner diversity index (*H*’) over each survey season. Significance denoted by alpha ‘*’ 0.05, ‘**’ 0.01, ‘***’ 0.001.

| **Site** | **Code** | ***S* (total)** | ***S* (ave)** | ***d*** | ***H*’** |
| --- | --- | --- | --- | --- | --- |
| Large remnant |  | 6 | 3.42±0.34 | 0.78±0.11 | 0.87±0.12 |
|  | LR1 | 7 |  | 1.37 | 1.45 |
|  | LR2 | 6 |  | 0.99 | 1.26 |
| Fragmented |  | 8 | 2.08±0.24*** | 0.57±0.08 | 0.47±0.10 |
|  | FR1 | 6 |  | 0.95 | 1.10 |
|  | FR2 | 3 |  | 0.58 | 0.82 |
|  | FR3 | 5 |  | 0.94 | 1.3 |
|  | FR4 | 2 |  | 0.38 | 0.52 |
| Adjacent |  | 8 | 3.82±0.36 | 0.79±0.11 | 0.80±0.13 |
|  | AFR1 | 7 |  | 1.17 | 1.47 |
|  | AFR2 | 7 |  | 1.15 | 1.09 |
| Restored |  | 6 | 3.27±0.36 | 0.77±0.11 | 0.64±0.13 |
|  | RS1 | 6 |  | 0.94 | 1.15 |
|  | RS2 | 4 |  | 0.68 | 1.22 |
| **Summer: *Banksia attenuata* flowering** | | | | | |
| Total |  |  | 2.54±0.25* |  |  |
| Large remnant |  | 5 | 2.83±0.50 | 0.54±0.13 | 0.64±0.13 |
|  | LR1 | 5 |  | 1.02 | 0.56 |
|  | LR2 | 5 |  | 0.84 | 0.80 |
| Fragmented |  | 7 | 1.92±0.32 | 0.55±0.13 | 0.30±0.12 |
|  | FR1 | 4 |  | 0.87 | 0.39 |
|  | FR2 | 2 |  | 0.91 | 0.19 |
|  | FR3 | 4 |  | 0.78 | 0.62 |
|  | FR4 | 1 |  | 0 | 0.05 |
| Adjacent |  | 7 | 3.60±0.22 | 0.76±0.06 | 0.93±0.11 |
|  | AFR1 | 6 |  | 1.17 | 0.80 |
|  | AFR2 | 5 |  | 0.91 | 0.62 |
| Restored |  | 5 | 2.60±0.46 | 0.87±0.22 | 0.50±0.19 |
|  | RS1 | 4 |  | 0.80 | 0.50 |
|  | RS2 | 3 |  | 1.03 | 0.12 |
| **Winter: *Banksia menziesii* flowering** | | | | | |
| Total |  |  | 3.27±0.24 |  |  |
| Large remnant |  | 6 | 4±0.33 | 1.03±0.07 | 1.10±0.09 |
|  | LR1 | 5 |  | 1.19 | 0.43 |
|  | LR2 | 6 |  | 1.32 | 0.61 |
| Fragmented |  | 5 | 2.25±0.36 | 0.54±0.11 | 0.56±0.13 |
|  | FR1 | 5 |  | 0.79 | 0.81 |
|  | FR2 | 3 |  | 0.69 | 0.25 |
|  | FR3 | 3 |  | 0.65 | 0.27 |
|  | FR4 | 1 |  | 0 | 0.14 |
| Adjacent |  | 8 | 4±0.58 | 0.82±0.16 | 0.69±0.19 |
|  | AFR1 | 5 |  | 0.87 | 0.88 |
|  | AFR2 | 7 |  | 1.3 | 0.74 |
| Restored |  |  | 3.83±0.28 | 0.70±0.07 | 0.75±0.16 |
|  | RS1 | 6 |  | 0.99 | 0.96 |
|  | RS2 | 4 |  | 0.69 | 0.73 |
