# Supplementary material for: Restored and remnant Banksia woodlands elicit different foraging behavior in avian pollinators
Source: Ecol Evol. 2021 Jul 27;11(17):11774–85. doi: 10.1002/ece3.7946 (PMC8427588; doi:10.1002/ece3.7946)
Supplement: Supplementary file 5 — Appendix S5 [file ECE3-11-11774-s009.docx]

**Appendix S5.**

**
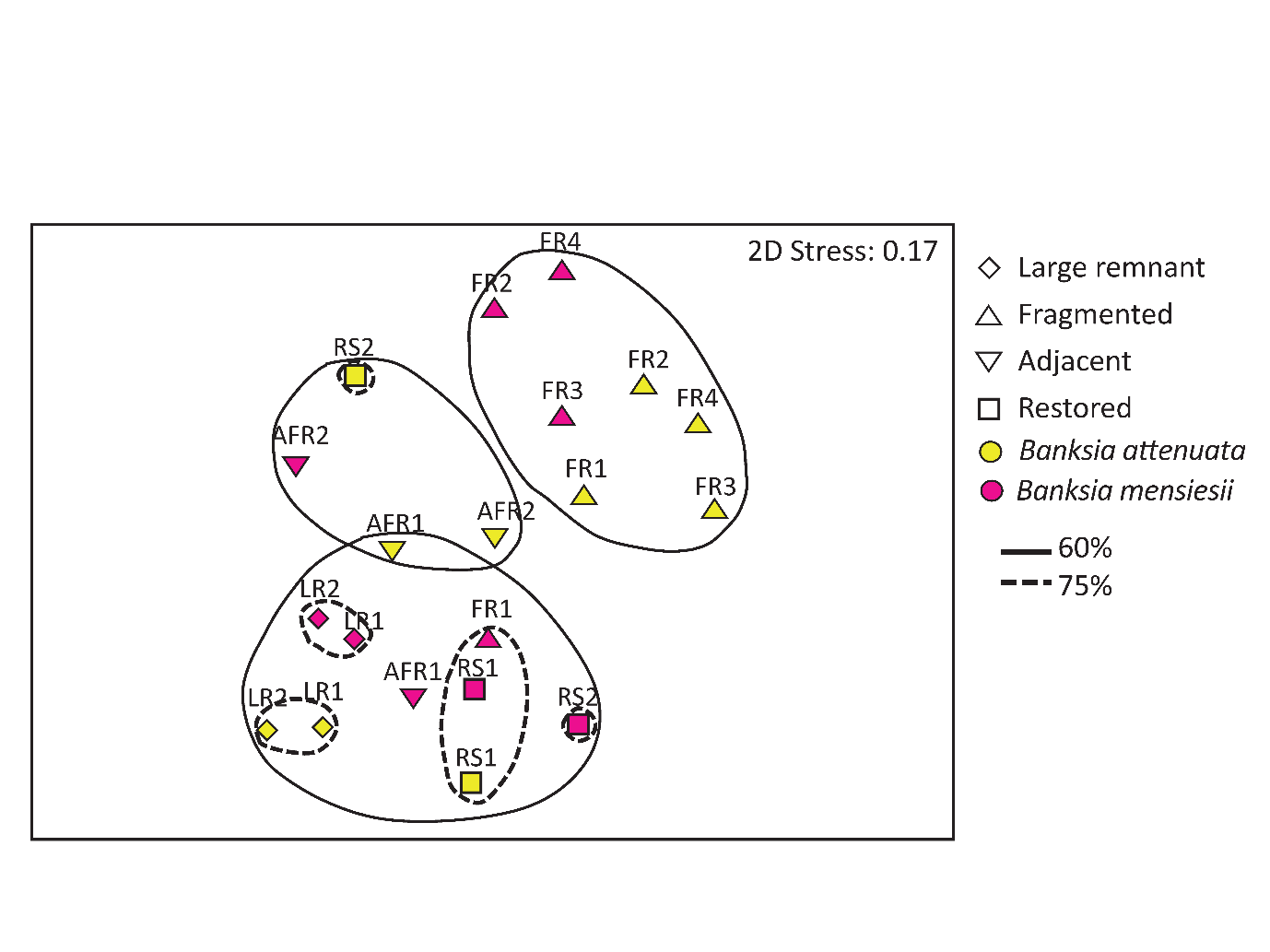
**

**Figure.** Non-metric multidimensional scaling (NMDS) plots (resemblance by Bray-Curtis similarity), showing clustering of the abundance of bird visitors from observed point counts for both flowering species *Banksia attenuata* and *B. menziesii*, with overlaid clusters at a similarity level of 60% (black line) and 75% (dashed line). Large remnants, LR1 and LR2; fragmented sites, FR1 - FR4; adjacent fragments, AFR1 and AFR2; and restored sites, RS1 and RS2.
