# Supplementary material for: Restored and remnant Banksia woodlands elicit different foraging behavior in avian pollinators
Source: Ecol Evol. 2021 Jul 27;11(17):11774–85. doi: 10.1002/ece3.7946 (PMC8427588; doi:10.1002/ece3.7946)
Supplement: Supplementary file 6 — Appendix S6 [file ECE3-11-11774-s007.docx]

**Appendix S6.** Overall relative abundance of floral visitors (observed probing an inflorescences) per site

| **Species** | **Large remnant** | **Fragmented** | **Adjacent** | **Restored** |
| --- | --- | --- | --- | --- |
| Australian Ringneck  (*Barnardius zonarius* subspecies *semitorquatus*) |  |  | 0.003 |  |
| Brown honeyeater  (*Lichmera indistincta*) | 0.459 | 0.171 | 0.249 | 0.123 |
| New Holland Honeyeater  (*Phylidonyris novae-hollandiae*) | 0.053 |  | 0.397 | 0.081 |
| Rainbow Lorikeet  (*Trichoglossus moluccanus*) |  | 0.003 | 0.011 |  |
| Red Wattlebird  (*Anthochaera carunculata*) |  | 0.268 | 0.051 |  |
| Silvereye (*Zosterops lateralis*) | 0.004 | 0.029 |  |  |
| Singing Honeyeater  (*Lichenostomus virescens*) | 0.028 | 0.023 |  |  |
| Western Spinebill  (*Acanthorhynchus superciliosus*) | 0.061 | 0.019 | 0.025 | 0.014 |
| Western Wattlebird  (*Anthochaera lunulata*) | 0.069 | 0.097 | 0.187 | 0.472 |
| White-cheeked honeyeater (*Phylidonyris niger*) | 0.329 | 0.390 | 0.076 | 0.306 |
