# Supplementary material for: Restored and remnant Banksia woodlands elicit different foraging behavior in avian pollinators
Source: Ecol Evol. 2021 Jul 27;11(17):11774–85. doi: 10.1002/ece3.7946 (PMC8427588; doi:10.1002/ece3.7946)
Supplement: Supplementary file 7 — Appendix S7 [file ECE3-11-11774-s001.docx]

**Appendix S7.**


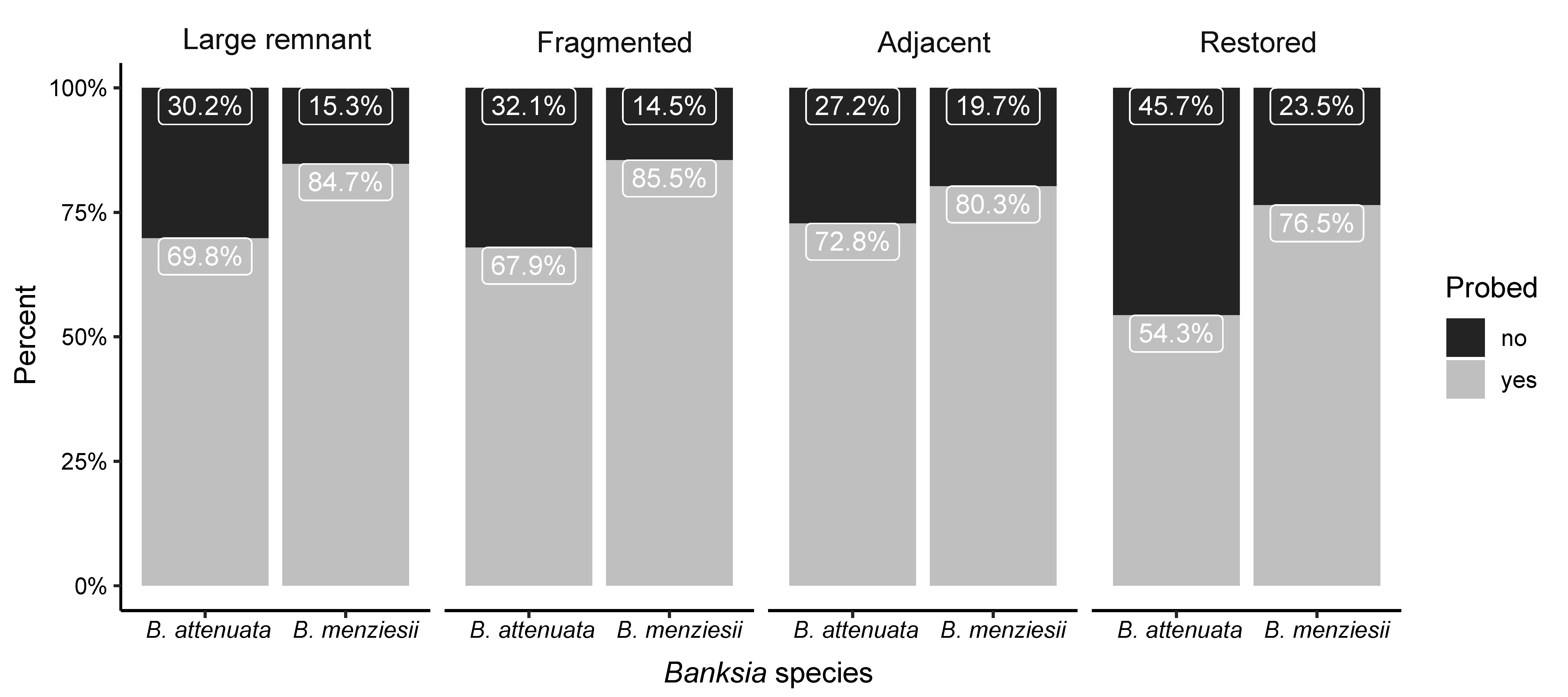
**Figure.** Percentage of observed inflorescence probing per *Banksia* species across site types.
