# Supplementary material for: Restored and remnant Banksia woodlands elicit different foraging behavior in avian pollinators
Source: Ecol Evol. 2021 Jul 27;11(17):11774–85. doi: 10.1002/ece3.7946 (PMC8427588; doi:10.1002/ece3.7946)
Supplement: Supplementary file 8 — Appendix S8 [file ECE3-11-11774-s010.docx]

**Appendix S8.** Percentage of foraging movements

| *Banksia* species | Site type | Intra-tree | Near-neighbour | Distant | Out of site | Non-*banksia* | out of sight | did not see leave | Total no. of movements |
| --- | --- | --- | --- | --- | --- | --- | --- | --- | --- |
| *Banksia attenuata* | Large remnant | 40.7 | 25.2 | 19.6 | 12.6 | 1.9 | 6.9 | 6.1 | 246 |
|  | Fragmented | 39.6 | 33.3 | 11.7 | 9.0 | 6.3 | 13.5 | 3.0 | 133 |
|  | Adjacent | 36.8 | 34.9 | 15.1 | 13.2 |  | 4.8 | 1.8 | 227 |
|  | Restored | 40.4 | 19.1 | 17.0 | 12.8 | 10.6 | 25.0 | 1.6 | 64 |
| *Banksia menziesii* | Large remnant | 43.8 | 28.7 | 28.7 | 3.8 | 1.1 | 22.7 |  | 343 |
|  | Fragmented | 47.5 | 41.4 | 10.0 | 1.1 |  | 25.3 | 0.5 | 752 |
|  | Adjacent | 40.2 | 36.9 | 18.2 | 3.4 | 1.3 | 19.8 | 1.0 | 667 |
|  | Restored | 35.3 | 47.5 | 14.1 | 1.6 | 1.6 | 26.9 | 1.0 | 707 |

Network metrics for bird foraging movement networks site, with observed edge weight diversity (*O*), and ꭕ^2^ analysis, lower matrix, Pearson’s chi-square value on the lower matrix and *P* value score on the upper matrix.

| Site type | *O* | Site | LR1 | LR2 | FR1 | FR2 | FR3 | FR4 | AFR1 | AFR2 | RS1 | RS2 |
| --- | --- | --- | --- | --- | --- | --- | --- | --- | --- | --- | --- | --- |
| Large Remnant | 0.73 | LR1 |  | n.s. | <0.01 | n.s. | <0.01 | <0.01 | <0.01 | <0.01 | <0.01 | <0.01 |
|  | 0.74 | LR2 | 6.52 |  | <0.01 | n.s. | 0.03 | <0.01 | <0.01 | <0.01 | 0.04 | <0.01 |
| Fragmented | 0.70 | FR1 | 35.60 | 29.65 |  | n.s. | 0.02 | 0.01 | <0.01 | n.s. | <0.01 | n.s. |
|  | 0.72 | FR2 | 7.03 | 4.23 | 2.53 |  | n.s. | <0.01 | n.s. | n.s. | n.s. | n.s. |
|  | 0.73 | FR3 | 18.21 | 10.77 | 11.55 | 2.53 |  | <0.01 | <0.01 | n.s. | n.s. | n.s. |
|  | 0.72 | FR4 | 28.40 | 23.77 | 14.98 | 13.80 | 12.96 |  | <0.01 | 0.01 | <0.01 | <0.01 |
| Adjacent | 0.74 | AFR1 | 22.64 | 21.74 | 51.44 | 8.19 | 21.20 | 24.48 |  | <0.01 | 0.01 | <0.01 |
|  | 0.71 | AFR2 | 22.72 | 16.25 | 1.29 | 0.86 | 5.72 | 14.60 | 28.49 |  | 0.01 | n.s. |
| Restored | 0.76 | RS1 | 20.52 | 10.09 | 25.76 | 4.43 | 5.32 | 22.65 | 14.47 | 14.11 |  | 0.04 |
|  | 0.68 | RS2 | 21.32 | 15.32 | 5.84 | 7.18 | 8.57 | 17.12 | 23.07 | 6.69 | 10.29 |  |
