# Supplementary material for: Restored and remnant Banksia woodlands elicit different foraging behavior in avian pollinators
Source: Ecol Evol. 2021 Jul 27;11(17):11774–85. doi: 10.1002/ece3.7946 (PMC8427588; doi:10.1002/ece3.7946)
Supplement: Supplementary file 9 — Appendix S9 [file ECE3-11-11774-s004.docx]

**Appendix S9.
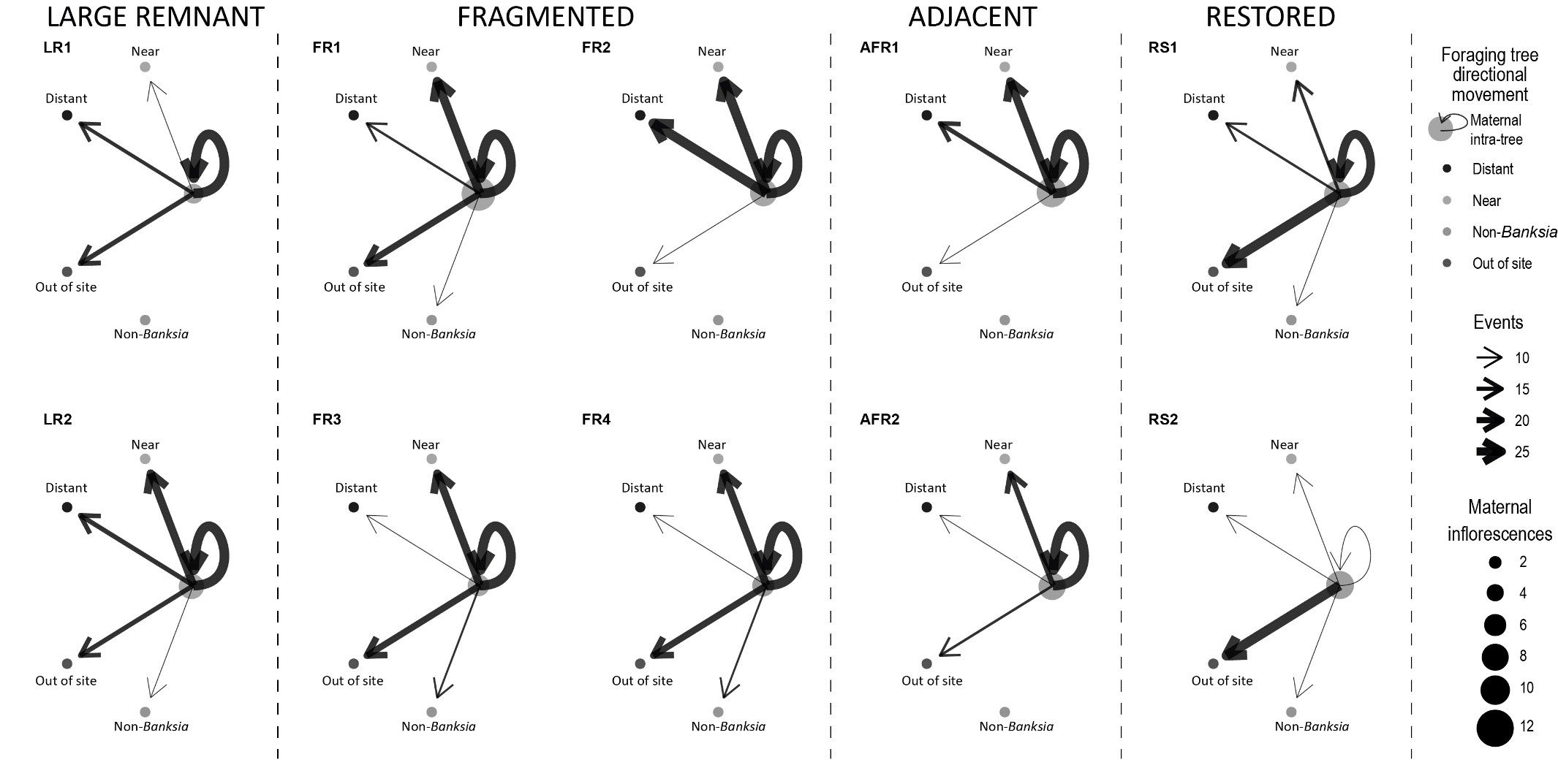
Figure. *Banksia attenuata* foraging movement network graphs:** Bird movements per site on *Banksia attenuata* as foraging movement network graphs. Events are total observations of movement after the initial probe foraging event. Nodes represent the location travelled after the initial foraging bout on the maternal tree; maternal tree node size indicates the average number of inflorescences on the observed tree; arrows indicate the directional movement after the first foraging bout and arrow width indicates the number of events observed.

**
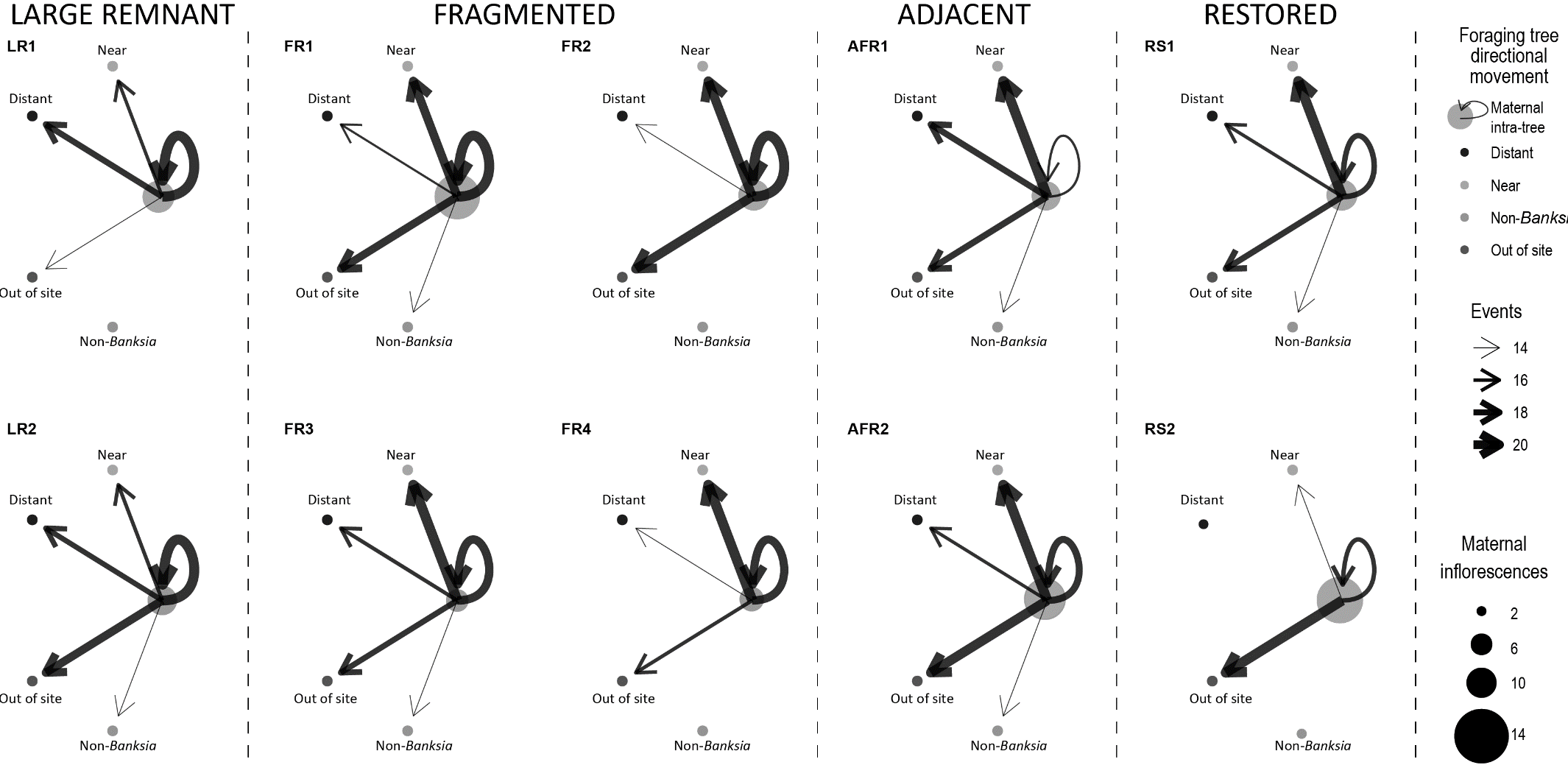
Figure. *Banksia menziesii* foraging movement network graphs:** Bird movements per site on *B. menziesii* visualized as foraging movement network graphs. Events are total observations of movement after the initial probe foraging event. Nodes represent the location travelled after the initial foraging bout on the maternal tree; maternal tree node size indicates the average number of inflorescences on the observed tree; arrows indicate the directional movement after the first foraging bout and arrow width indicates the number of events observed.
