# Supplementary material for: Restored and remnant Banksia woodlands elicit different foraging behavior in avian pollinators
Source: Ecol Evol. 2021 Jul 27;11(17):11774–85. doi: 10.1002/ece3.7946 (PMC8427588; doi:10.1002/ece3.7946)
Supplement: Supplementary file 10 — Appendix S10 [file ECE3-11-11774-s006.docx]

**Appendix S10.** Congruence contingency tables between bird interaction networks per site type and per site.

| Site type | | | Large remnant | | | Fragmented | | | | Adjacent | | | Restored | | |
| --- | --- | --- | --- | --- | --- | --- | --- | --- | --- | --- | --- | --- | --- | --- | --- |
| Large remnant | | |  | | |  | | | |  | | |  | | |
| Fragmented | | | 0.004 | | |  | | | |  | | |  | | |
| Adjacent | | | n.s | | | 0.001 | | | |  | | |  | | |
| Restored | | | n.s | | | n.s. | | | | n.s. | | |  | | |
| Site type | Site | LR1 | | LR2 | FR1 | | FR2 | FR3 | FR4 | | AFR1 | AFR2 | | RS1 | RS2 |
| Large Remnant | LR1 |  | |  |  | |  |  |  | |  |  | |  |  |
|  | LR2 | <0.05 | |  |  | |  |  |  | |  |  | |  |  |
| Fragmented | FR1 | <0.01 | | <0.05 |  | |  |  |  | |  |  | |  |  |
|  | FR2 | n.s. | | n.s. | n.s. | |  |  |  | |  |  | |  |  |
|  | FR3 | n.s. | | n.s. | n.s. | | n.s. |  |  | |  |  | |  |  |
|  | FR4 | n.s. | | n.s. | n.s. | | n.s. | n.s. |  | |  |  | |  |  |
| Adjacent | AFR1 | <0.01 | | n.s. | <0.01 | | n.s. | n.s. | n.s. | |  |  | |  |  |
|  | AFR2 | n.s. | | n.s. | n.s. | | n.s. | n.s. | n.s. | | n.s. |  | |  |  |
| Restored | RS1 | <0.05 | | n.s. | n.s. | | n.s. | n.s. | n.s. | | <0.05 | n.s. | |  |  |
|  | RS2 | <0.05 | | n.s. | <0.01 | | n.s. | n.s. | n.s. | | n.s. | n.s. | | n.s. |  |

Network metrics for bird interaction networks per site type.

| Site type | Site | Nodes | Edges | Edge density | Density | *O* | Eigen centrality value | Centrality degree | Components |
| --- | --- | --- | --- | --- | --- | --- | --- | --- | --- |
| Large Remnant | LR1 | 16 | 8 | 0.031 | 0.029 | 0.84 | 5.33 | 0.14 | 11 |
|  | LR2 | 11 | 3 | 0.025 | 0.011 | 0.49 | 5.50 | 0.14 | 9 |
| Fragmented | FR1 | 16 | 4 | 0.016 | 0.015 | 0.69 | 3.30 | 0.09 | 15 |
|  | FR2 | 4 | 2 | 0.125 | 0.007 | 0.44 | 2.25 | 0.44 | 3 |
|  | FR3 | 7 | 8 | 0.163 | 0.029 | 0.84 | 4.60 | 0.26 | 4 |
|  | FR4 | 3 | 0 | 0.000 | 0.000 | 0 | 0.00 | 0.00 | 3 |
| Adjacent | AFR1 | 10 | 7 | 0.070 | 0.018 | 0.83 | 5.61 | 0.22 | 7 |
|  | AFR2 | 13 | 5 | 0.030 | 0.018 | 0.43 | 6.73 | 0.15 | 10 |
| Restored | RS1 | 8 | 5 | 0.078 | 0.018 | 0.19 | 16.00 | 0.39 | 4 |
|  | RS2 | 6 | 4 | 0.111 | 0.015 | 0.19 | 20.21 | 0.32 | 4 |
